# MRSA Infection in the Thigh Muscle Leads to Systemic Disease, Strong Inflammation, and Loss of Human Monocytes in Humanized Mice

**DOI:** 10.3389/fimmu.2022.892053

**Published:** 2022-06-20

**Authors:** Sophia Hung, Liane Dreher, Joachim Diessner, Stefan Schwarz, Knut Ohlsen, Tobias Hertlein

**Affiliations:** ^1^ Institute for Molecular Infection Biology, University of Würzburg, Würzburg, Germany; ^2^ Institute of Microbiology and Epizootics, Centre for Infection Medicine, Department of Veterinary Medicine, Freie Universität Berlin, Berlin, Germany; ^3^ Veterinary Centre for Resistance Research (TZR), Department of Veterinary Medicine, Freie Universität Berlin, Berlin, Germany; ^4^ Department of Obstetrics and Gynaecology, University Hospital of Würzburg, Würzburg, Germany

**Keywords:** humanized mice, MRSA - methicillin-resistant *Staphylococcus aureus*, monocyte, bacterial infection model, inflammation, NSG, staphylocccal infection/epidemiology

## Abstract

MRSA (Methicillin-resistant *Staphylococcus aureus*) is the second-leading cause of deaths by antibiotic-resistant bacteria globally, with more than 100,000 attributable deaths annually. Despite the high urgency to develop a vaccine to control this pathogen, all clinical trials with pre-clinically effective candidates failed so far. The recent development of “humanized” mice might help to edge the pre-clinical evaluation closer to the clinical situation and thus close this gap. We infected humanized NSG mice (huNSG: (NOD)-*scid IL2R_γ_^null^* mice engrafted with human CD34+ hematopoietic stem cells) locally with *S. aureus* USA300 LAC* *lux* into the thigh muscle in order to investigate the human immune response to acute and chronic infection. These mice proved not only to be more susceptible to MRSA infection than wild-type or “murinized” mice, but displayed furthermore inferior survival and signs of systemic infection in an otherwise localized infection model. The rate of humanization correlated directly with the severity of disease and survival of the mice. Human and murine cytokine levels in blood and at the primary site of infection were strongly elevated in huNSG mice compared to all control groups. And importantly, differences in human and murine immune cell lineages surfaced during the infection, with human monocyte and B cell numbers in blood and bone marrow being significantly reduced at the later time point of infection. Murine monocytes in contrast behaved conversely by increasing cell numbers. This study demonstrates significant differences in the *in vivo* behavior of human and murine cells towards *S. aureus* infection, which might help to sharpen the translational potential of pre-clinical models for future therapeutic approaches.

## Introduction

*Staphylococcus aureus* is one of the most successful pathogens of our time and an efficient colonizer, which can be transmitted in community and healthcare. It causes a wide range of infections from skin and soft tissue infections to life-threatening diseases like pneumonia, endocarditis and bacteremia (1, 2). Its high genetic variability and flexibility as well as its infamous feature of developing or acquiring antibiotic resistance makes this pathogen a major problem for our healthcare systems and causes high costs and many deaths every year. Its Methicillin-resistant variant (MRSA) is the second-most leading cause of attributable deaths to antibiotic-resistant bacteria world-wide (3).

Despite the undeniable and urgent need for new therapeutics, particularly for a vaccine, the development has been hampered by poor correlation of pre-clinical and clinical efficacy (4, 5). Two explanations have been proposed for this discrepancy since most pre-clinical studies were performed in mouse models: (i) *S. aureus* is well known for a long list of virulence factors like bi-component toxins, surface proteins and immunomodulators, that act with different efficiency against human and murine immune components (6–8) and (ii) the human’s and the mouse’s immune system react differently to an encounter with *S. aureus* (9).

In consequence, the main idea to resolve this problem has been to adapt the pre-clinical models in order to become more representative for the clinical situation in humans. “Humanized mice” – mice with a human immune system – might be a first step to meet these needs. The most widespread type of humanized mice is the NSG ((NOD)-*scid IL2R_γ_^null^*) mouse engrafted with human (CD34+) hematopoietic stem cells (huNSG), which constitute a human immune system in this mouse (10, 11). A limited number of earlier studies about *S. aureus* infection in humanized mice have already proven, that these mice are more susceptible to infection with this pathogen (12–15). Since most of these studies focused on the short-term or local effects of *S. aureus* challenge in humanized mice, we wondered how the human immune cells adapt during a longer period of infection and whether we can see differences emerge between the murine and the human immune response over the course of infection.

To this end, we infected humanized mice locally in the thigh muscle with *S. aureus* and monitored the local course of infection as well as the systemic spreading of the bacteria. Furthermore, we correlated the rate of humanization in these mice with the severity of infection. Finally, in an effort to examine the immune system’s struggle between fighting the pathogen on one hand and limiting the inflammation on the other hand, we characterized the recruitment of human and murine immune cells from/to bone marrow, spleen and blood, as well as the repertoire of human and murine cytokines in blood and the infected thigh muscle.

## Material And Methods

### Ethics Statement

All animal studies were approved by the local government of Lower Franconia, Germany (approval numbers 55.2-2532-2-836 and 55.2-2532-2-1129) and performed in strict accordance with the guidelines for animal care and experimentation of the German Animal Protection Law and the DIRECTIVE 2010/63/EU of the EU. The animals were housed in cages under standardized lighting conditions and had *ad libitum* access to food and water. All *in vivo* imaging was performed under isoflurane anesthesia, and all efforts were made to minimize suffering. All experimentations with anonymized and non-traceable human cord blood were approved by the ethics committee of the University Würzburg.

### Humanization and Murinization Procedure (Incl. CD34+ Isolation)

Female and male NSG (NOD.Cg-Prkdc^scid^ Il2rg^tm1Wjl^/SzJ) mice from the Jackson Laboratories (Bar Harbor, ME, USA) or female Balb/c mice (BALB/cJRj, Janvier labs, Le Genest-Saint-Isle, France) were used for all experiments. Human CD34+ hematopoietic stem cells were isolated from human cord blood by magnetic separation (EasySep™ Human Cord Blood CD34 Positive Selection Kit II, STEMCELL technologies, Cologne, Germany) following the manufacturer’s protocol and purity controlled by flow cytometry. Humanized NSG (huNSG) mice were generated by engraftment of 100,000 hCD34+ cells (of a donor mix) at the age of 6 – 8 weeks, similar to the procedures described in earlier publications (15–18). Briefly, mice received whole-body irradiation with a sub-lethal dose of 2 Gy and hematopoietic stem cells were administered intravenously 2 hours later. For the generation of murinized NSG mice (muNSG), mice were treated as the huNSG mice, but received 100,000 bone marrow cells from a Balb/c donor instead of human CD34+ cells. The peripheral blood of huNSG mice was analyzed for the presence and frequency of murine CD45+, as well as human CD45+, CD3+ and CD20+ cells at week 18 post engraftment by flow cytometry.

### Thigh Infection Model (Incl. Bacterial Burden)

HuNSG mice were infected at 18 weeks post engraftment. Therefore, the left thigh of each mouse was shaved, disinfected and 1 x 10^8^ CFU of bioluminescent *S. aureus* LAC* *lux* (19) was injected into the muscle as described before (20). The infection dose was generated from an overnight shaking culture (at 37°C) in B medium by pelleting the bacteria, resuspending them and diluting them to the final concentration in sterile 0.9% NaCl solution. Age-matched muNSG, wild-type NSG and Balb/c mice served as controls to investigate differences during the course of infection. The wellbeing of each mouse was inspected and scored every 12 hours p.i. and the weight measured every 24 hours. Mice were either sacrificed at day 2 or at day 7 p.i. with the exception of seven huNSG mice and one wild-type NSG mouse that reached the humane end point of the experiment prematurely. At each end point, we harvested the infected thigh muscle, kidneys, liver, spleen and heart, as well as peripheral blood and bone marrow from tibia and femur from each mouse. The thigh muscle, kidneys, liver and heart were homogenized in 0.9% NaCl and serial dilutions were plated on B agar plates in order to determine the bacterial burden. The spleens were homogenized by pressing through a 70 µm filter with 0.9% NaCl. Serial dilutions of this cell homogenate were then plated on B agar plates to determine the bacterial burden. Cell suspensions from the bone marrow were harvested by flushing both femurs and tibias of each mouse with 0.9% NaCl solution.

### *In Vivo* Bioluminescence Imaging (BLI)

All infected mice were monitored by *in vivo* Bioluminescence Imaging (BLI) throughout the course of infection as described earlier (21). Briefly, starting at 5 min p.i. and followed by 24 h inspection intervals, the luminescence signal of each mouse was measured from dorsal view with a Lumina II bioluminescence imager (PerkinElmer, Waltham, MA, USA). The signal of the infected thigh muscle was measured by LivingImage 3.2 software (PerkinElmer, Waltham, MA, USA) within a region of interest with same geometry and size for each mouse and time point (imaging settings: exposure, 120 s; FStop, 1; excitation, block; emission, open; FOV, D; height, 1.5 cm).

### Flow Cytometry

All flow cytometric analyses were performed and analyzed on a MACSQuant flow cytometer (Miltenyi Biotec, Bergisch Gladbach, Germany). Human CD34+ hematopoietic stem cell purity was checked by staining the isolated cells with anti-human CD34 and anti-human CD3 antibodies. Cells were only used for humanization, when purity was > 85% CD34+ cells of all cells and CD3 T cells < 1% of all cells. At the end of the humanization period, the peripheral blood was stained after red blood cell lysis with anti-mouse CD45 and anti-human CD45/CD3/CD19 antibodies to monitor the rate of humanization (which was hCD45+ cells/(hCD45+ cells + mCD45+ cells)). Spleen cell suspensions were stained after red blood cell lysis with anti-human CD45/CD3/CD19 antibodies, bone marrow cell suspensions with anti-human CD45/CD14/CD66b/CD19 antibodies and peripheral blood at the end of the infection experiments with combinations of anti-human CD45/CD3/CD4/CD8/CD19/CD14/CD66b and anti-mouse CD45/Ly6C/Ly6G antibodies. The gating strategy is outlined in **Figure S4**. All antibodies were supplied by Miltenyi Biotec (Bergisch Gladbach, Germany).

### Determination of Cytokine Levels and Myeloperoxidase Activity

For determination of cytokine levels and myeloperoxidase activity, the thigh muscle homogenate was centrifuged at 3,000 x g for 5 minutes and the supernatant stored at -80°C until further processing. The peripheral blood samples were agglutinated overnight at 4°C, then centrifuged at 15,000 x g for 15 minutes, the serum harvested and stored at -80°C until cytokine measurement. Levels of human or murine MCP-1, IL-1β, IL-6, IL-10, IL-17A, IFN-γ and TNF-α in the infected thigh muscles or peripheral blood were measured with custom-mixed Luminex assays from Bio-Techne (Wiesbaden, Germany) following the manufacturer’s manual. As internal cross-reactivity controls, we applied samples of the cytokine/chemokine standards of the mouse cytokine kit for the measurements with the human cytokine kit and vice versa, but could only detect neglectable cross-reactivity. Myeloperoxidase activity in the infected thigh muscle was measured with small adaptions as described earlier (13). Briefly, 50 µL supernatant of an 1.25% dilution of thigh muscle homogenate in PBS was mixed 1:1 with 3,3’,5,5’-tetramethylbenzidine (TMB) (Thermo Fisher Scientific, Waltham, MA, USA) and incubated for 15 minutes at room temperature. The reaction was stopped by adding another 50 µL 2N H_2_SO_4_ to the reaction and the absorbance at 450 nm determined with a microplate reader. Results are expressed as arbitrary units (AU).

## Results

### Humanized NSG Mice Are More Susceptible to Localized Infection With MRSA

Since a limited number of earlier studies *of S. aureus* infection in humanized mice showed higher susceptibility (12–15), we wondered whether the severity of *S. aureus* infection in our deep-tissue abscess model is increased, too. Therefore, we humanized 6 – 8 weeks old NSG mice by the administration of human cord-blood isolated CD34+ hematopoietic stem cells. After 18 weeks of engraftment, we checked the frequency and number of human CD45+ cells in the blood and included only mice with more than 10% human CD45+ blood cells among all CD45+ cells (humanization rate) into the infection experiments. The average humanization rate for the mice applied in this study was 25.1% (range: 10.9 – 54.5%). We applied three control groups: wild-type NSG mice, wild-type Balb/c mice, which are regularly used to investigate *S. aureus* infection, and murinized NSG (muNSG) mice, which served to exclude a possible influence of the humanization process on the outcome of the infection experiments. The wild-type NSG mouse group was not irradiated since we found no difference in the number of murine neutrophils or monocytes at 18 weeks post irradiation in a pilot experiment (with n = 3), which was in accordance with earlier results by others (22). The murinized mice were generated by the same procedure as humanized mice but received murine bone marrow cells (from Balb/c mice) instead of human CD34+ hematopoietic stem cells. This engraftment resulted in the establishment of a murine instead of a human immune system in otherwise immunodeficient NSG mice.

**Figure 1:**
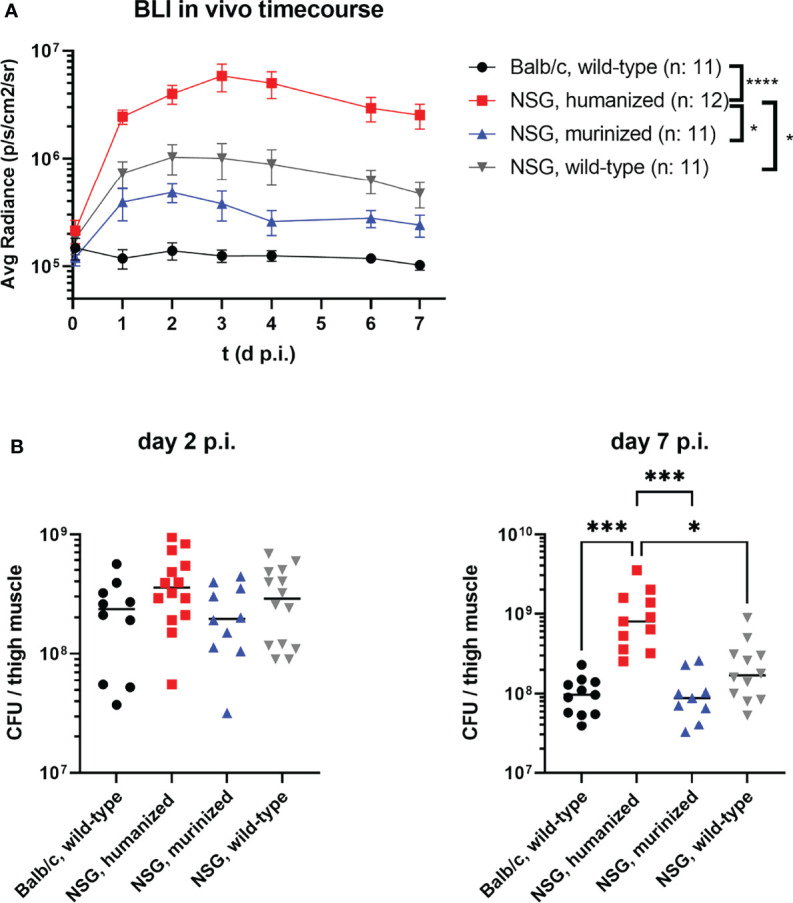
The course of MRSA thigh muscle infection in humanized and control mouse groups. Mice were infected with 1x10^8^ CFU *S. aureus* LAC* *lux* into the left thigh muscle. **(A)** Bioluminescence signal of the luciferase-expressing bacteria at the primary site of infection (thigh muscle) was measured every 24 hours. Depicted are the mean radiance levels +/- SEM for each respective group. Statistical significance was tested for each time point with Kruskal-Wallis test. **(B)** The infected thigh muscles were recovered at either day 2 or day 7 p.i., homogenized and plated in serial dilutions on agar plates to determine the bacterial burden. Shown are the individual colony-forming-units (CFU) per mouse and the corresponding medians per group. Statistical significance was determined by Kruskal-Wallis with Dunn’s post-test (*p ≤ 0.05, ***p ≤ 0.001, ****p ≤ 0.0001).

Applying bioluminescence imaging on a daily basis during the *S. aureus* infection allowed to monitor the bacterial burden and local spreading during the course of the infection (exemplary mice are depicted in **Figure S1**). The humanized mouse group showed a significantly higher signal intensity than all other groups as early as 24 hours p.i. and remained significantly stronger throughout the experiment (**Figure 1A**).

This observation was confirmed by the bacterial burden in the infected thigh muscle (**Figure 1B**). The infected thigh muscles of HuNSG showed an almost 10-fold higher bacterial load at day 7 p.i. compared to all other groups, including immunodeficient wt NSG mice. Of particular interest in this regard is, that while all other mouse groups were able to reduce or control the number of bacteria in the infected thigh, the number of bacteria in the HuNSG group increased significantly (p = 0.0187) between day 2 and day 7 p.i. and was at both time points higher than the initial inoculum of 10^8^ CFU.

### Impaired Survival of HuNSG After Localized Infection and Signs of Systemic Disease

**Figure 2:**
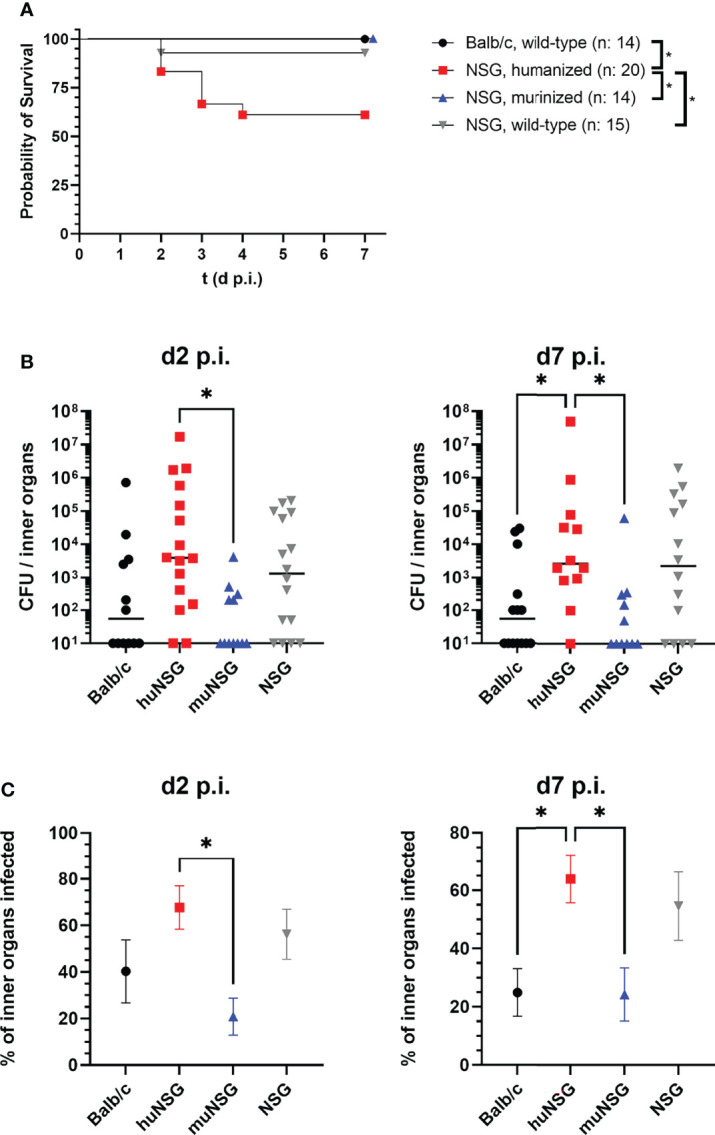
Mortality of the locally infected mice, signs of systemic infection and humanization rates of the humanized mice groups. **(A)** Seven of 20 humanized mice and one of 14 wild-type NSG mice reached the humane end point during the course of infection. These mice were removed from the experiment and counted as “dead”. Statistically significant differences between the groups were tested with Log-rank (Mantel-Cox) test. **(B)** The spleen, heart, liver and kidneys were recovered at the indicated time points and the bacterial burden determined. Displayed are the individual values per mouse and the corresponding median per group. **(C)** The percentage of tested inner organs (kidneys, liver, spleen and heart) with bacterial burden per animal was calculated and the mean +/- SEM per group is displayed (n: huNSG = 12; NSG, wild-type = 14; Balb/c = 14; muNSG = 11). Statistical significance was tested with Kruskal-Wallis with Dunn’s post-test **(B, C)** (*p ≤ 0.05).

The wellbeing of each individual mouse was judged daily based on a score sheet throughout the whole infection experiment and score points assigned accordingly. The score sheet included physical signs of disease like weight loss (**Figure S2**) or heavy breathing and behavioral signs like reduced activity or hunchback posture. The only signs of disease (besides the abscess formation) encountered in all mice except the huNSG group and one wild-type NSG mouse was a moderate loss of weight, especially in the first two days after infection (**Figure S2**). The huNSG group, in contrast, lost weight continuously and heavily during the whole course of infection and showed additionally severe signs of disease. In consequence, 7 of 20 mice from the huNSG group (and one wild-type NSG mouse) reached the humane end point, which defined the maximum of animal suffering that is acceptable for our experimental purpose. Since these mice had to be sacrificed and removed from the experiment, we designated them as “dead” and plotted them in a survival plot to visualize the impact of localized thighmuscle infection with *S. aureus* for each group (**Figure 2A**). The huNSG mouse group showed significantly inferior survival to all other mouse groups which raised the question, how local *S. aureus* infection in the thigh muscle could cause this dramatic escalation of disease.

A possible explanation for this severity of disease might be spreading of bacteria from the local site of infection to inner organs, thus causing systemic infection. This would be particularly interesting since the applied *S. aureus* USA300 derivate is notorious for causing skin and soft tissue infections, but also for systemic, life-threatening infections (1, 23).

In order to test this hypothesis, we investigated the bacterial burden in liver, kidneys, heart and spleen of all mice. The bacterial colonization of these organs was highly diverse and showed no clear picture for each organ at its own, but gave a distinct pattern, when all investigated inner organs were assumed as one combined system. Adding the numbers of all bacteria in these organs showed a significantly higher bacterial burden in the inner organs of huNSG mice compared to Balb/c and muNSG group mice (**Figure 2B**). Only the wild-type NSG mouse group showed similarly high numbers of systemic bacteria. However, not only the total number of bacteria in the tested inner organs was higher for huNSG and NSG mice, the percentage of infected inner organs was also significantly elevated (**Figure 2C**).

### The Stronger the Humanization Rate the Higher the Chance to Succumb to Infection

**Figure 3:**
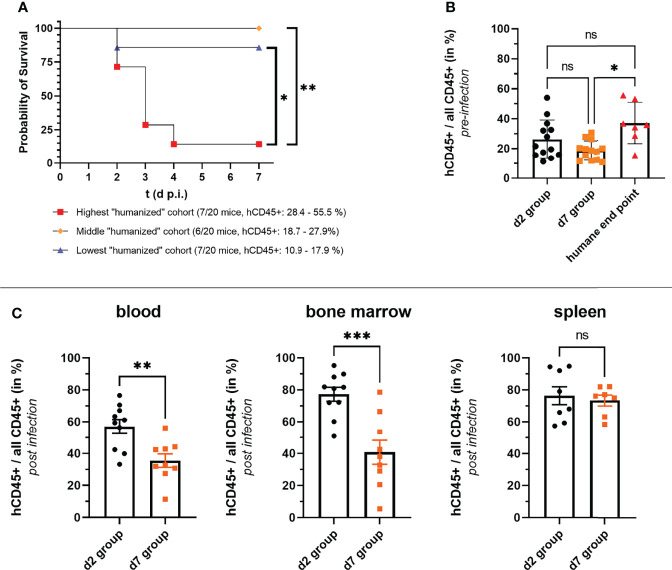
Correlation of rate of humanization (hCD45+ cells/all CD45+ cells) and severity of infection. **(A, B)** The numbers of human and murine CD45+ cells were determined by flow cytometry before mice were infected with *S. aureus*. **(A)** HuNSG mice which either reached the humane end point of the experiment or were assigned to the day 7 p.i. group were ordered dependent on their rate of humanization into three groups. All but one mouse that reached the humane end point was in the group with the highest rate of humanization as depicted in the survival graph. Statistical significance was tested with the Log-rank (Mantel-Cox) test. **(B)** The rate of humanization of each huNSG mice in the experiment is depicted according to the outcome/end point of the experiment. Surviving mice were either assigned to the day 2 or day 7 p.i. end point groups, while the seven huNSG mice which reached the humane end point prematurely between day 2 and day 7 p.i. were assigned to the “humane end point group”. **(C)** The rate of humanization at the end of the infection experiment was additionally measured by flow cytometry for the d2 and d7 groups in blood, spleen and bone marrow. Statistical significance was tested by Kruskal-Wallis with Dunn’s post-test (ns, not significant, *p ≤ 0.05, **p ≤ 0.01, ***p ≤ 0.001).

The finding, that humanized mice succumbed to infection following a local challenge with *S. aureus* was rather surprising, and exceeded our first hypothesis of humanized mice being more susceptible than non-humanized ones considerably. We wondered whether we could more precisely determine the factors which caused this increased susceptibility and shift from local to systemic infection. The first question we asked in this regard was whether the rate of humanization (hCD45+/(hCD45+ and mCD45+) could serve as a predictor for the severity of infection within the humanized mouse group. Those humanized mice, which were either assigned to the day 7 p.i. group or reached the humane end point during the experiment, were, therefore, grouped into three groups dependent on their rate of humanization prior to infection. And indeed, the group with the highest rate of humanization prior to infection included significantly more mice which reached the humane end point within 7 days post thigh muscle challenge than the other two groups (**Figure 3A**). The rate of humanization was thus from a retrospective point of view a very good predictor for a humanized mouse’s chance of survival.

But also reviewing our data from the end-point perspective, namely is there a difference in the rate of humanization between mice which survived until day 7 p.i. (d7 group) and those that reached the humane end point, led to a similar interpretation: the mice that succumbed to infection had an overall higher rate of humanization (prior to infection) than those that survived (**Figure 3B**). Of note, there was no difference in the rate of humanization prior to infection between the day 2 p.i. group (d2 group) and the d7 group or the huNSG that reached the humane end point.

The rate of humanization in blood, spleen and bone marrow was in addition measured at the end point of the d2 and d7 groups. Notably, the mice from the d7 group had a significantly lower humanization rate than the d2 group mice in blood and bone marrow at their respective end points (**Figure 3C**), although their blood humanization rates prior infection were similar (**Figure 3A**). No differences between both groups could be seen in the spleen.

### Stronger Inflammation in HuNSG

Since the presence of human immune cells in huNSG was prominently linked to a more severe infection, we asked next, whether there are differences in the human and murine immune response and inflammation. First, we checked whether the myeloperoxidase activity, a main component of neutrophils to combat bacterial infection, was different between the huNSG mice and the control groups in the infected thigh muscle. Since we found no differences in the levels of myeloperoxidase activity between the groups or time points (**Figure S3**), we focused on the next question which was whether the pattern or the levels of human or murine cytokines/chemokines were different.

**Figure 4:**
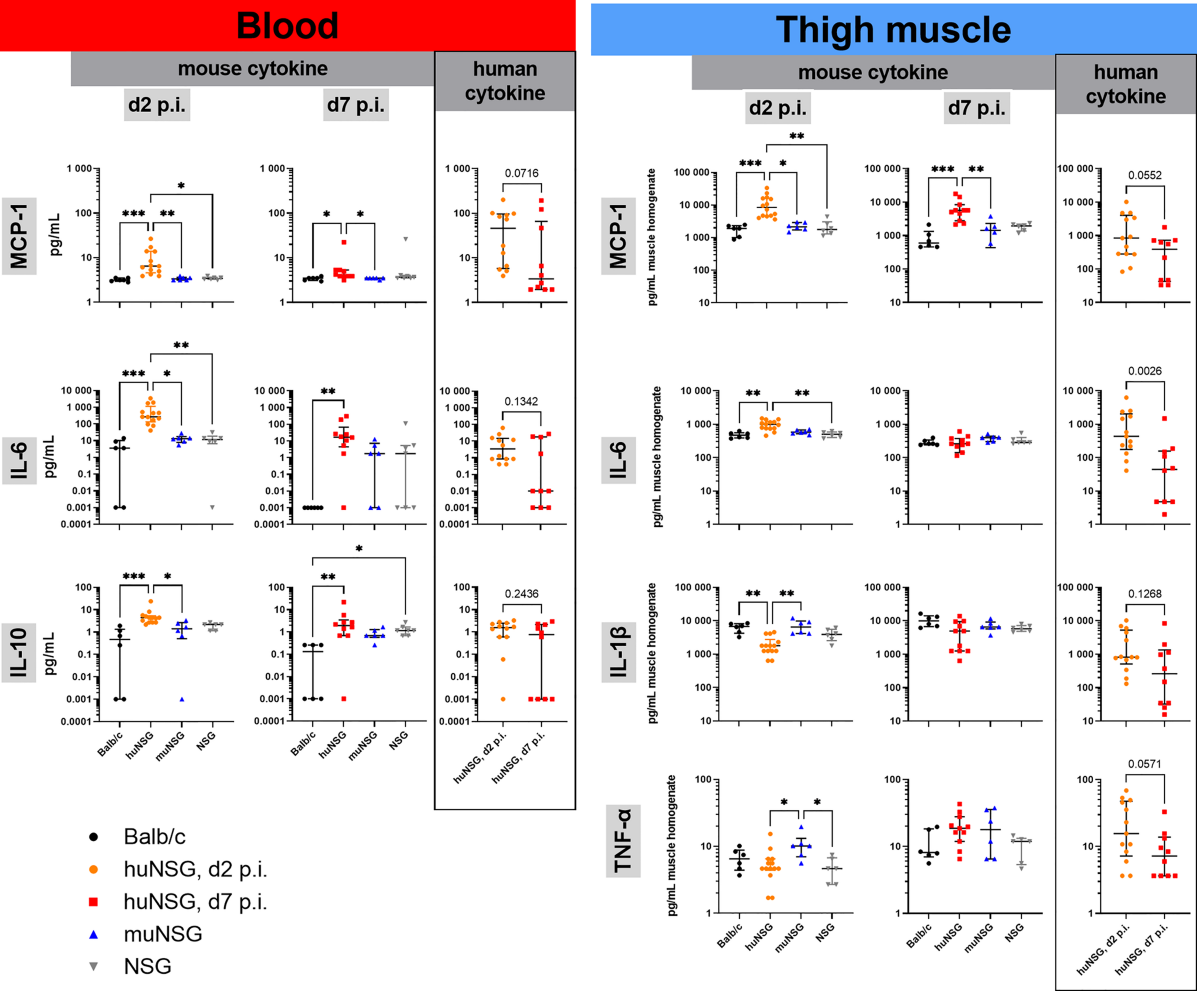
Cytokine levels in blood and infected thigh muscle at early and late time point of infection. Displayed are the individual levels per mouse and the median with interquartile range for each respective group/time point. Levels in blood serum or muscle homogenate were determined with customized Luminex panels. Statistical significance was tested by Kruskal-Wallis with Dunn’s post-test for the murine cytokines or Mann-Whitney-test for the human counterparts (*p ≤ 0.05, **p ≤ 0.01, ***p ≤ 0.001).

Therefore, the levels of human and murine MCP-1, IL-1β, IL-6, IL-10, IL-17A, IFN-γ and TNF-α in the blood and the infected thigh muscle were measured at days 2 and 7 p.i. The levels of all tested signaling molecules was in general lower in the blood than at the site of infection (**Figure 4**). The levels of IL-17A and IFN-γ were in all groups either below or at the detection limit. The huNSG mice delivered overall higher levels of murine MCP-1, IL-6 and IL-10 in the blood than the other groups and had in addition similarly high levels of their human counterparts in the blood, too. It is important to note in this regard, that purified human cytokines/chemokines delivered only neglectable signal in the murine Luminex assay and vice versa. The pattern was similar, albeit at much higher levels, for MCP-1 and IL-6 in the infected thigh muscle (**Figure 4**). The huNSG mice showed higher levels than all other groups and had in addition human cytokines/chemokines at similar levels than murine ones. The levels of murine IL-1β and TNF-α was slightly lower for the huNSG mice, but the levels were in similar range when their human counterparts were added. The levels of murine MCP-1, IL-10 and IL-1β were in both, blood and muscle at similar levels at day 2 and day 7 p.i., while the level of IL-6 decreased. A completely different pattern could be seen for the human cytokines/chemokines of the huNSG mice. Human MCP-1, IL-1β, IL-6 and IL-10 were all lower at day 7 compared to day 2 p.i. (**Figure 4**), thus indicating a clearly different behavior of the human and the murine immune system, even within the chimeric huNSG mice.

### Levels of B and T Cells at Early and Late Infection

**Figure 5:**
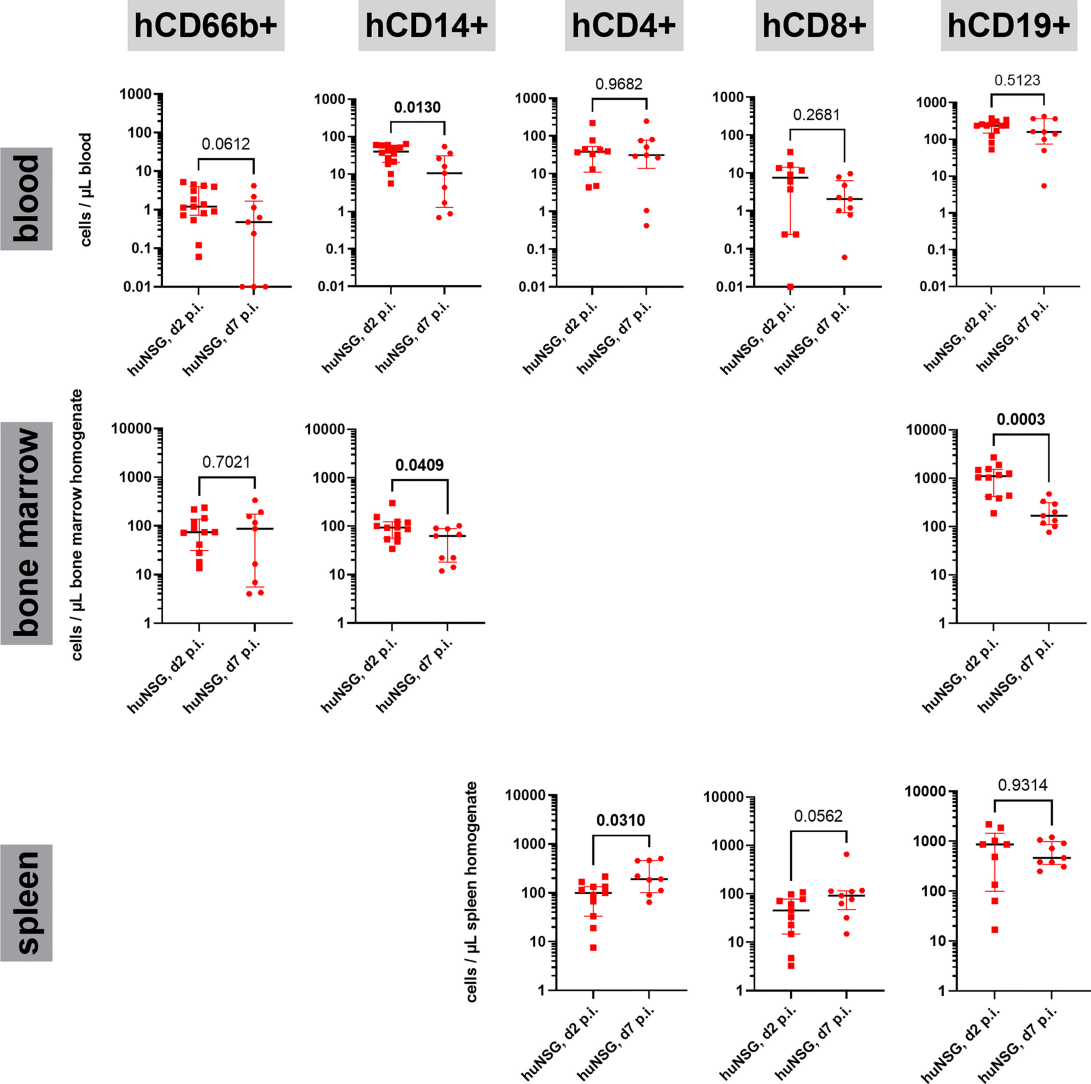
Human immune cell lineages in blood, bone marrow and spleen during the course of thigh infection. Cells were first gated for expression of hCD45 and lack of mCD45 expression, then for the respective lineage markers (hCD66b: granulocytes, hCD14: monocytes, hCD4: T helper cells, hCD8: cytotoxic T cells, CD19: B cells). The gating strategy can be found in **Figure S4**. Statistical significance was tested with Mann-Whitney-test and p-values displayed (significant values (p < 0.05) are depicted as bold numbers).

This difference in the signaling of human and murine immune systems raised the question whether the immune cell lineages behave differently, too? Since the dominating fraction of the human immune system in huNSG mice consists of T and B cells, we first focused on their numbers in blood, spleen and bone marrow. Since we already knew from the determination of the rate of humanization in the blood, that the surviving huNSG mice (d7 group) showed a lower rate of humanization at day 7 than the d2 group (see **Figure 3C**), we assumed that their prevalence might be lower in the blood at day 7 p.i. However surprisingly, we saw no differences in their overall cell numbers (**Figure 5**). The number of human CD19+ B cells in the bone marrow was significantly lower at day 7 than at day 2 p.i., but unchanged in the spleen. The T cell numbers on the opposite increased in the spleen between both time points. The increase of T cell numbers in the spleen indicates the induction of a T cell response against *S. aureus* as indicated by earlier publications (15).

### Decreased Number of Human CD14+ Monocytes in the Blood of Humanized Mice at Later Stage Infection

Since neither the human T nor the human B cell compartment showed differences in their numbers in the blood of huNSG at day 2 and day 7 p.i., we wondered how other immune cell lineages reacted to the challenge with *S. aureus* in the thigh muscle and the development of systemic disease in the humanized mice. We checked the number of neutrophils (CD66b+) and monocytes (CD14+) in the blood and bone marrow. Neutrophil numbers were low in the blood at both time points, with a tendency towards lower numbers at day 7 p.i. Total numbers resembled those of earlier studies in huNSG mice (16). Monocytes on the opposite showed a very distinct pattern for blood and bone marrow. In both organs, the number of monocytes was significantly lower at day 7 than at day 2 p.i. (**Figure 5**). This became even more puzzling when we investigated the numbers of murine monocytes (Ly6C+ Ly6G-) in both, humanized and non-humanized mice for comparison (**Figure S5**). In all investigated mouse groups, the number of murine monocytes increased strongly and significantly between both time points, proving a strong activation. Even in huNSG mice, in which the number of human monocytes dropped severely, the number of murine monocytes increased strongly, reversing the ratio between both from day 2 to day 7 p.i. The drop in blood humanization rates between day 2 and day 7 p.i. (**Figure 3C**) can thus be explained by both, an increase of murine cells and the simultaneous decrease of human monocytes in the blood of huNSG mice.

## Discussion

Among the emerging threat posed by antibiotic-resistant bacteria, MRSA holds the pole position as the pathogen with most clinical cases and attributable deaths in the USA, and second in the European Union (24, 25). However, although there is a high urgency and need for novel therapies, many pre-clinically efficient approaches targeting *S. aureus* failed in clinical trials (4, 26). This lack of translational power from pre-clinical models to infection in humans is, at least to some extent, due to the pronounced host tropism of clinical *S. aureus* strains (9). Several ideas to overcome this obstacle have been proposed by the scientific community, with humanized mice being one of the most promising solutions (5, 7).

The basis for humanized mice was the development of highly immunodeficient mouse strains, that lack B, T, NK cells and support human haemato-lymphopoiesis after engraftment with human CD34+ stem cells (27, 28). Humanized NSG mice (huNSG) are the most widely used model in this context and have so far been used to study *S. aureus* in peritonitis, skin infection, pneumonia and osteomyelitis models (12–15). It became obvious in this limited number of recent studies, that huNSG are more susceptible to *S. aureus* than wild-type or “murinized” mice. We wondered how the human immune system adapts to long-term infection with MRSA and whether differences between the murine and the human immune response might surface which might explain this higher susceptibility?

We decided to investigate the behavior of the human immune system during infection with MRSA in the thigh muscle, which resembles a localized, deep-tissue abscess formation and covers both, acute and chronic phases of infection. We found in accordance with the earlier studies of staphylococcal infection in humanized mice, that they were more susceptible to bacterial infection resulting in higher bacterium-derived bioluminescence signal and higher bacterial burden at the primary site of infection. The bacterial load in the infected thigh muscle was significantly higher in huNSG mice than in murinized or wild-type NSG mice, as well as compared to Balb/c mice.

The most stunning observation, nonetheless, was that 35% of the humanized mice reached the humane end point between day 2 and day 7 p.i. (based on the animal welfare score sheet criteria) and had to be removed from the experiment due to critical signs of disease and infection. This was in contrast to all control groups in which only 1 wild-type NSG mouse reached this point. And even those humanized mice that survived until day 7 p.i. showed a much higher probability of bacterial spreading from the primary site of infection to the inner organs. When we took a closer look at the correlation of the rate of humanization and the severity of infection, it became clear, that the mice with a high rate of humanization (prior infection) had a strongly reduced chance of surviving local MRSA challenge compared to their less humanized littermates.

This divergence in the outcome of localized *S. aureus* infection between humanized and non-humanized mice asked the question which factors or components are responsible. We approached this question by investigating the immune system’s signaling and cell population dynamics during the infection and found significant differences between the human and the murine immune response. First, the cytokine and chemokine levels in huNSG mice were much higher than in the other groups, and we could detect both, human and murine cytokines in these mice. The higher levels of MCP-1, IL-6 and IL-10 suggested a much stronger inflammatory response to the bacterial challenge in the humanized mice, especially at day 2 p.i.

It is well known that several factors of *S. aureus*, that can result in pro-inflammatory cytokine signaling, are much more efficient against human cells than against their murine counterparts, like bi-component toxins, superantigens/enterotoxins or immunomodulators (6, 29–31).

However, in discrepancy to the increase of the bacterial burden in the thigh muscle and the inner organs, we could see lower levels of human cytokines at the later time point of infection. Reasons for this might be either the regulation of the immune response respectively the inflammation or an exhaustion of the immune response or its deployed cells. Either way, it might indicate the failing of the immune system to overcome the bacterial infection and thus explain the decreased survival of the huNSG mice.

Our hypothesis was that if it is exhaustion that leads to the reduced inflammatory status and the impaired survival of the huNSG mouse group, we would see the depletion or reduction of at least one major lineage of human immune cells. In order to investigate this, we took a closer look at the cell numbers of immune cell lineages in the blood, bone marrow and spleen by flow cytometry. We could detect considerable numbers of human B and T cells, as well as monocytes, but only very low numbers of human neutrophils in the blood, as described in an earlier study (16). We observed furthermore, as described for *S. aureus* osteomyelitis infection in huNSG mice (15), a significant increase of human T cell numbers in the spleen, which indicates a pathogen-induced human immune cell response in the animals. On the other hand, we found that the number of CD19+ B cells in the bone marrow decreased dramatically, while their numbers in blood and spleen remained stable. In addition, the numbers of CD14+ monocytes dropped similarly drastically in both, blood and bone marrow, which in turn might be the reason for the lower cytokine levels in blood and thigh muscle at the later time point. Both observations indicate that the human immune system in the huNSG mice gets caught in the maelstrom of local infection and bacterial spreading without being able to reproduce enough immune cells to overcome this challenge. This might, at least, in parts be based on the fact, that some human immune cell lineages are underrepresented in huNSG mice, most notably neutrophils (16), or that others are not terminally differentiated. At the first glance this might be regarded as a drawback of humanized mice in preclinical research for infectious disease. However, from a different point of view, it might even reflect the specific clinical need much better, in which success of therapy is important at most: in patients with an impaired immune response, particularly when neutrophil function is affected like congenital neutropenia or chronic granulomatous disease (32, 33).

The drop of human monocytes and B cells raises additionally the question about the influence of *S. aureus*. Is it simply the overwhelming presence of the bacteria that leads to the depletion of these cell lineages or is *S. aureus* actively depleting these cells by the action of virulence factors? Particularly the differences between human and murine monocytes are striking in this regard. While the numbers of human monocytes are fading away systemically during the course of infection, we could, in huNSG and control mice, measure an increase of the murine ones. Importantly, the fate of the human monocytes remains elusive at this point: future studies are necessary to unravel whether they are migrating to the site of infection, are undergoing apoptosis, or having their reproduction in the bone marrow affected. Furthermore, the dynamics of human and murine immune cells in the infected thigh muscle apart from the cytokine levels were inaccessible in this study, too, and should be investigated on a single cell level in future in order to understand the shift from local to systemic infection. Considering, that several virulence factors of *S. aureus* show higher affinity or activity towards human than murine immune cells *in vitro*, like bi-component toxins, superantigens or immunomodulators (6, 29, 34), it seems reasonable to assume, that humanized mice are indeed representing the clinical course of infection more closely than wild-type mice. On the other hand, limitations of humanized NSG mice like weak human myeloid reconstitution and the lack of human thymus tissue (10, 35) have to be considered when interpreting the observed results in our infection model.

In summary, we could show in this study, that a local infection with MRSA in humanized mice induced systemic spreading of the bacteria and high mortality, while the infection stayed locally in non-humanized mice. We furthermore unraveled grave differences between the human and murine immune response against *S. aureus* in this *in vivo* system, particularly in the cytokine response and the recruitment of monocytes. These results might help to develop better disease-related pre-clinical models for the validation of novel therapeutic approaches and the identification of promising targets.

## Data Availability Statement

The original contributions presented in the study are included in the article/**Supplementary Material**. Further inquiries can be directed to the corresponding author.

## Ethics Statement

The animal study was reviewed and approved by local government of Lower Franconia, Germany (approval numbers 55.2-2532-2-836 and 55.2-2532-2-1129).

## Author Contributions

KO, TH, SS, and JD contributed to conception and design of the study. SH, LD, and TH performed the experiments. SH, LD, and TH analyzed the data. TH and SH wrote the first draft of the manuscript. KO, JD, and SS wrote sections of the manuscript. All authors contributed to manuscript revision, read, and approved the submitted version.

## Funding

We wish to thank the “Deutsche Forschungsgemeinschaft” (DFG OH97/8-1) for financial support. This publication was supported by the Open Access Publication Fund of the University of Wuerzburg.

## Conflict of Interest

The authors declare that the research was conducted in the absence of any commercial or financial relationships that could be construed as a potential conflict of interest.

## Publisher’s Note

All claims expressed in this article are solely those of the authors and do not necessarily represent those of their affiliated organizations, or those of the publisher, the editors and the reviewers. Any product that may be evaluated in this article, or claim that may be made by its manufacturer, is not guaranteed or endorsed by the publisher.

## References

[1] Turner NA Sharma-Kuinkel BK Maskarinec SA Eichenberger EM Shah PP Carugati M. Methicillin-Resistant *Staphylococcus aureus*: An Overview of Basic and Clinical Research. Nat Rev Microbiol (2019) 17(4):203–18. doi: 10.1038/s41579-018-0147-4 PMC6939889 30737488

[2] Lee AS de Lencastre H Garau J Kluytmans J Malhotra-Kumar S Peschel A. Methicillin-Resistant *Staphylococcus aureus* . Nat Rev Dis Primers (2018) 4:18033. doi: 10.1038/nrdp.2018.33 29849094

[3] Murray CJL Ikuta KS Sharara F Swetschinski L Aguilar GR Gray A. Antimicrobial Resistance Collaborators. Global Burden of Bacterial Antimicrobial Resistance in 2019: A Systematic Analysis. Lancet (2022) 399(10325):629–55. doi: 10.1016/S0140-6736(21)02724-0 PMC8841637 35065702

[4] Miller LS Fowler VG Shukla SK Rose WE Proctor RA. Development of a Vaccine Against *Staphylococcus aureus* Invasive Infections: Evidence Based on Human Immunity, Genetics and Bacterial Evasion Mechanisms. FEMS Microbiol Rev (2020) 44(1):123–53. doi: 10.1093/femsre/fuz030 PMC7053580 31841134

[5] Clegg J Soldaini E McLoughlin RM Rittenhouse S Bagnoli F Phogat S. *Staphylococcus aureus* Vaccine Research and Development: The Past, Present and Future, Including Novel Therapeutic Strategies. Front Immunol (2021) 12:705360. doi: 10.3389/fimmu.2021.705360 34305945 PMC8294057

[6] Koymans KJ Vrieling M Gorham RD Jr van Strijp JAG. Staphylococcal Immune Evasion Proteins: Structure, Function, and Host Adaptation. Curr Top Microbiol Immunol (2017) 409:441–89. doi: 10.1007/82_2015_5017 26919864

[7] Parker D. Humanized Mouse Models of *Staphylococcus aureus* Infection. Front Immunol (2017) 8:512. doi: 10.3389/fimmu.2017.00512 28523002 PMC5415562

[8] de Jong NWM van Kessel KPM van Strijp JAG. Immune Evasion by *Staphylococcus aureus* . Microbiol Spectr (2019) 7(2):GPP3-0061-2019. doi: 10.1128/microbiolspec.GPP3-0061-2019 PMC11590434 30927347

[9] Salgado-Pabón W Schlievert PM. Models Matter: The Search for an Effective *Staphylococcus aureus* Vaccine. Nat Rev Microbiol (2014) 12(8):585–91. doi: 10.1038/nrmicro3308 24998740

[10] Shultz LD Brehm MA Garcia-Martinez JV Greiner DL. Humanized Mice for Immune System Investigation: Progress, Promise and Challenges. Nat Rev Immunol (2012) 12(11):786–98. doi: 10.1038/nri3311 PMC3749872 23059428

[11] Ito R Takahashi T Ito M. Humanized Mouse Models: Application to Human Diseases. J Cell Physiol (2018) 233(5):3723–8. doi: 10.1002/jcp.26045 28598567

[12] Knop J Hanses F Leist T Archin NM Buchholz S Gläsner J. *Staphylococcus aureus* Infection in Humanized Mice: A New Model to Study Pathogenicity Associated With Human Immune Response. J Infect Dis (2015) 212(3):435–44. doi: 10.1093/infdis/jiv073 25657257

[13] Tseng CW Biancotti JC Berg BL Gate D Kolar SL Müller S. Increased Susceptibility of Humanized NSG Mice to Panton-Valentine Leukocidin and *Staphylococcus aureus* Skin Infection. PloS Pathog (2015) 11(11):e1005292. doi: 10.1371/journal.ppat.1005292 26618545 PMC4664407

[14] Prince A Wang H Kitur K Parker D. Humanized Mice Exhibit Increased Susceptibility to *Staphylococcus aureus* Pneumonia. J Infect Dis (2017) 215(9):1386–95. doi: 10.1093/infdis/jiw425 PMC5853420 27638942

[15] Muthukrishnan G Wallimann A Rangel-Moreno J Bentley KLM Hildebrand M Mys K. Humanized Mice Exhibit Exacerbated Abscess Formation and Osteolysis During the Establishment of Implant-Associated *Staphylococcus aureus* Osteomyelitis. Front Immunol (2021) 12:651515. doi: 10.3389/fimmu.2021.651515 33815412 PMC8012494

[16] Coughlan AM Harmon C Whelan S O'Brien EC O'Reilly VP Crotty P. Myeloid Engraftment in Humanized Mice: Impact of Granulocyte-Colony Stimulating Factor Treatment and Transgenic Mouse Strain. Stem Cells Dev (2016) 25(7):530–41. doi: 10.1089/scd.2015.0289 26879149

[17] Baird A Deng C Eliceiri MH Haghi F Dang X Coimbra R. Mice Engrafted With Human Hematopoietic Stem Cells Support a Human Myeloid Cell Inflammatory Response *In Vivo* . Wound Repair Regen (2016) 24(6):1004–14. doi: 10.1111/wrr.12471 PMC5161601 27663454

[18] Tothova Z Krill-Burger JM Popova KD Landers CC Sievers QL Yudovich D. Multiplex CRISPR/Cas9-Based Genome Editing in Human Hematopoietic Stem Cells Models Clonal Hematopoiesis and Myeloid Neoplasia. Cell Stem Cell (2017) 21(4):547–55.e8. doi: 10.1016/j.stem.2017.07.015 28985529 PMC5679060

[19] Umstätter F Domhan C Hertlein T Ohlsen K Mühlberg E Kleist C. Vancomycin Resistance Is Overcome by Conjugation of Polycationic Peptides. Angew Chem Int Ed Engl (2020) 59(23):8823–7. doi: 10.1002/anie.202002727 PMC7323874 32190958

[20] Hertlein T Sturm V Kircher S Basse-Lüsebrink T Haddad D Ohlsen K. Visualization of Abscess Formation in a Murine Thigh Infection Model of *Staphylococcus aureus* by 19F-Magnetic Resonance Imaging (MRI). PloS One (2011) 6(3):e18246. doi: 10.1371/journal.pone.0018246 21455319 PMC3063824

[21] Hertlein T Sturm V Lorenz U Sumathy K Jakob P Ohlsen K. Bioluminescence and 19F Magnetic Resonance Imaging Visualize the Efficacy of Lysostaphin Alone and in Combination With Oxacillin Against *Staphylococcus aureus* in Murine Thigh and Catheter-Associated Infection Models. Antimicrob Agents Chemother (2014) 58(3):1630–8. doi: 10.1128/AAC.01422-13 PMC3957871 24366730

[22] Walcher L Müller C Hilger N Kretschmer A Stahl L Wigge S. Effect of Combined Sublethal X-Ray Irradiation and Cyclosporine A Treatment in NOD Scid Gamma (NSG) Mice. Exp Anim (2019) 68(1):1–11. doi: 10.1538/expanim.18-0056 30078790 PMC6389519

[23] Tenover FC Goering RV. Methicillin-Resistant *Staphylococcus aureus* Strain USA300: Origin and Epidemiology. J Antimicrob Chemother (2009) 64(3):441–6. doi: 10.1093/jac/dkp241 19608582

[24] Centers for Disease Control and Prevention. Antibiotic Resistance Threats in the United States (2019). Atlanta, GA: Centers for Disease Control and Prevention. Available at: https://www.cdc.gov/drugresistance/pdf/threats-report/2019-ar-threats-report-508.pdf (Accessed January 19, 2022).

[25] Cassini A Högberg LD Plachouras D Quattrocchi A Hoxha A Simonsen GS. Attributable Deaths and Disability-Adjusted Life-Years Caused by Infections With Antibiotic-Resistant Bacteria in the EU and the European Economic Area in 2015: A Population-Level Modelling Analysis. Lancet Infect Dis (2019) 19(1):56–66. doi: 10.1016/S1473-3099(18)30605-4 30409683 PMC6300481

[26] Fowler VG Jr Proctor RA. Where Does a *Staphylococcus aureus* Vaccine Stand? Clin Microbiol Infect (2014) 20 Suppl 5(0 5):66–75. doi: 10.1111/1469-0691.12570 24476315 PMC4067250

[27] Ishikawa F Yasukawa M Lyons B Yoshida S Miyamoto T Yoshimoto G. Development of Functional Human Blood and Immune Systems in NOD/SCID/IL2 Receptor {Gamma} Chain(Null) Mice. Blood (2005) 106(5):1565–73. doi: 10.1182/blood-2005-02-0516 PMC1895228 15920010

[28] Shultz LD Lyons BL Burzenski LM Gott B Chen X Chaleff S. Human Lymphoid and Myeloid Cell Development in NOD/LtSz-Scid IL2R Gamma Null Mice Engrafted With Mobilized Human Hemopoietic Stem Cells. J Immunol (2005) 174(10):6477–89. doi: 10.4049/jimmunol.174.10.6477 15879151

[29] Spaulding AR Salgado-Pabón W Kohler PL Horswill AR Leung DY Schlievert PM. Staphylococcal and Streptococcal Superantigen Exotoxins. Clin Microbiol Rev (2013) 26(3):422–47. doi: 10.1128/CMR.00104-12 PMC3719495 23824366

[30] DuMont AL Yoong P Day CJ Alonzo F 3rd McDonald WH Jennings MP. *Staphylococcus aureus* LukAB Cytotoxin Kills Human Neutrophils by Targeting the CD11b Subunit of the Integrin Mac-1. Proc Natl Acad Sci U S A (2013) 110(26):10794–9. doi: 10.1073/pnas.1305121110 PMC3696772 23754403

[31] Lambris JD Ricklin D Geisbrecht BV. Complement Evasion by Human Pathogens. Nat Rev Microbiol (2008) 6(2):132–42. doi: 10.1038/nrmicro1824 PMC2814840 18197169

[32] Amulic B Cazalet C Hayes GL Metzler KD Zychlinsky A. Neutrophil Function: From Mechanisms to Disease. Annu Rev Immunol (2012) 30:459–89. doi: 10.1146/annurev-immunol-020711-074942 22224774

[33] Winkelstein JA Marino MC Johnston RB Jr Boyle J Curnutte J Gallin JI. Chronic Granulomatous Disease. Report on a National Registry of 368 Patients. Med (Baltimore) (2000) 79:155–69. doi: 10.1097/00005792-200005000-00003 10844935

[34] Spaan AN Vrieling M Wallet P Badiou C Reyes-Robles T Ohneck EA. The Staphylococcal Toxins γ-Haemolysin AB and CB Differentially Target Phagocytes by Employing Specific Chemokine Receptors. Nat Commun (2014) 5:5438. doi: 10.1038/ncomms6438 25384670 PMC4228697

[35] Lee JY Han AR Lee DR. T Lymphocyte Development and Activation in Humanized Mouse Model. Dev Reprod (2019) 23(2):79–92. doi: 10.12717/DR.2019.23.2.079 31321348 PMC6635618

