# Supplementary material for: MRSA Infection in the Thigh Muscle Leads to Systemic Disease, Strong Inflammation, and Loss of Human Monocytes in Humanized Mice
Source: Front Immunol. 2022 Jun 20;13:892053. doi: 10.3389/fimmu.2022.892053 (PMC9251014; doi:10.3389/fimmu.2022.892053)
Supplement: Supplementary file 1 [file DataSheet_1.pdf]

Supplementary Material

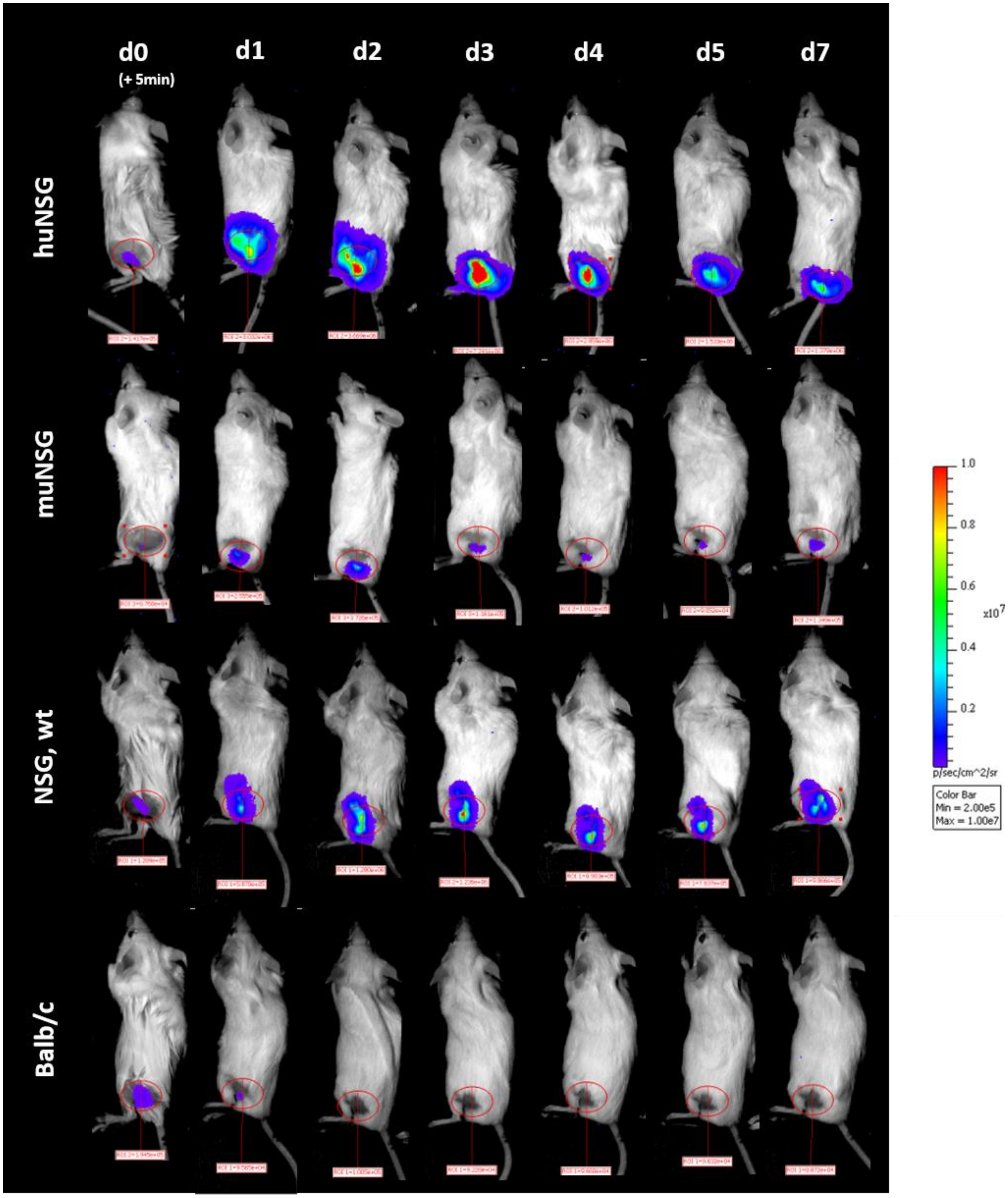

Figure S1: Bioluminescent overlay images of one representative mouse per group during the course of infection. The region-of-interest (ROI) had the same size and geometry for each mouse.

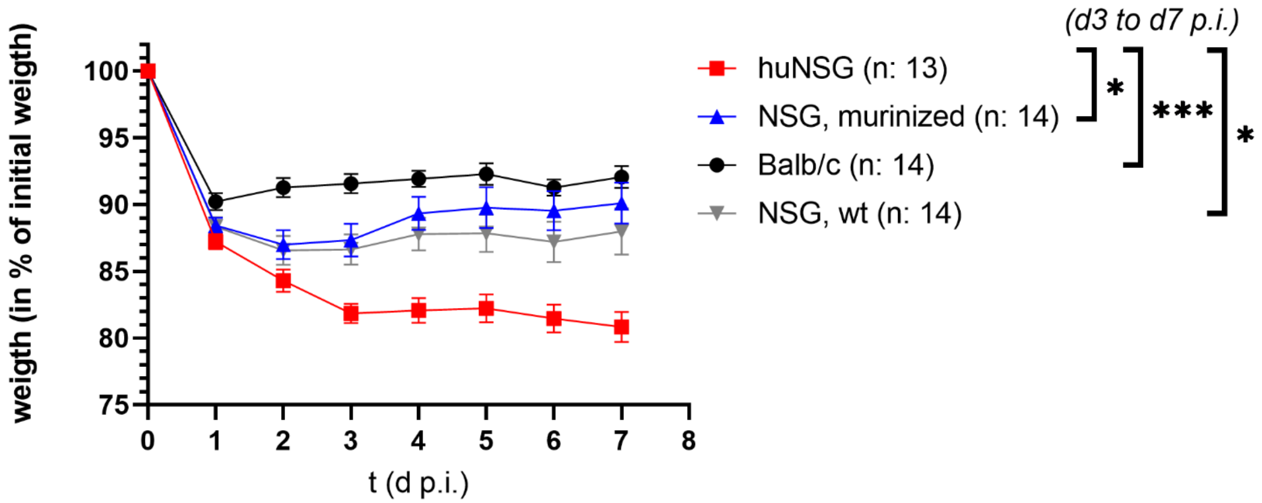

Figure S2: Weight loss during the course of infection. Visualized are the mean values +/- SEM per group of all mice which survived until day 7 p.i.. Statistical significance was tested for each time point with Kruskal-Wallis test.

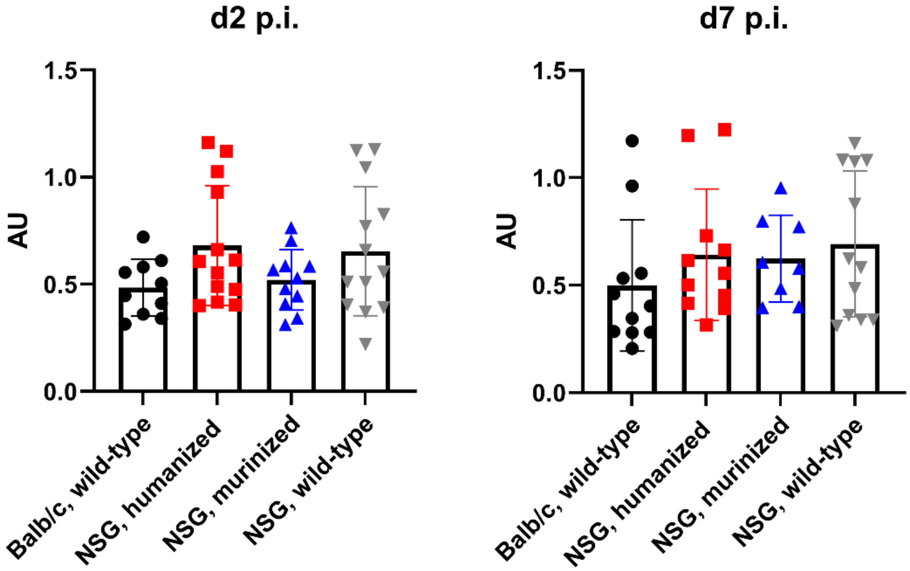

Figure S3: Myeloperoxidase activity in the infected thigh muscle. Infected thigh muscles were recovered at day 2 and 7 p.i., homogenized and the myeloperoxidase activity measured in a 1.5% dilution of this homogenate. (AU: arbitrary units)

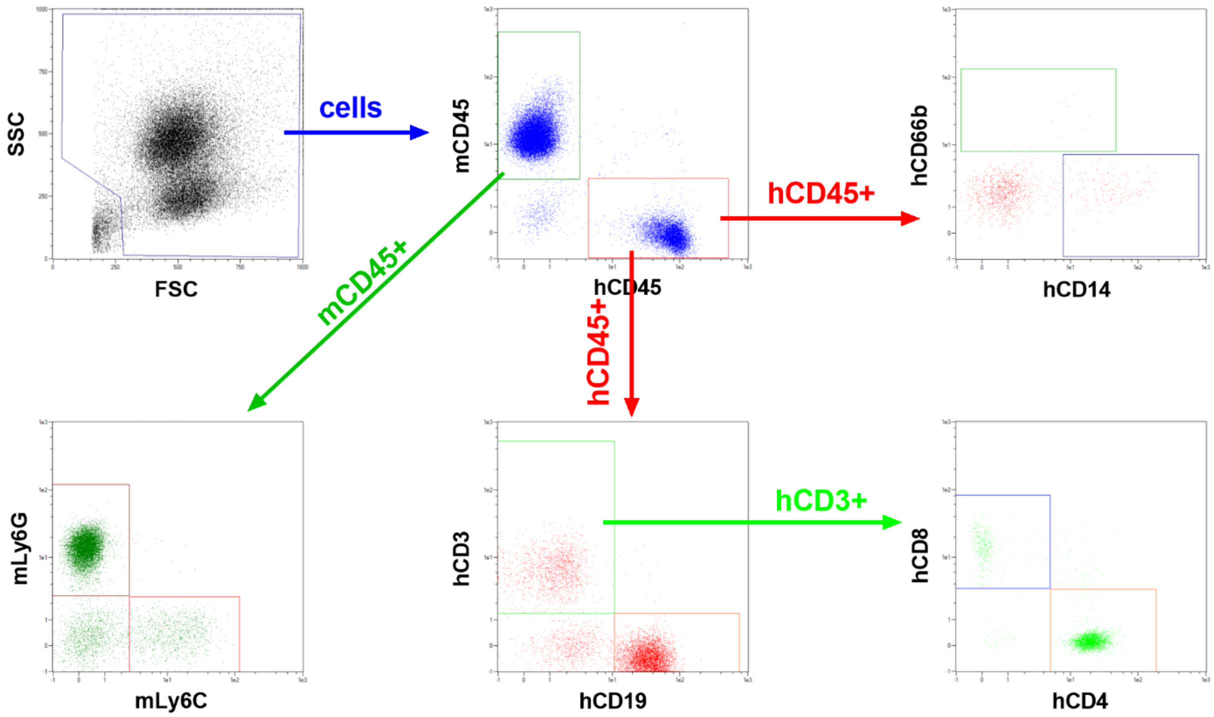

Figure S4: Principal gating strategy for this study. Blood samples and spleen or bone marrow cell suspensions were stained with different combinations of antibodies as stated in the methods section. In general, we first excluded cell debris and then gated for human or murine cells based on species-specific CD45+ expression. The immune cell lineage was then determined for these gated cells based on their expression of following markers: human: CD66b: neutrophils, CD14: monocytes, CD3, CD4 or CD8: T cells, CD19: B cells; or mouse: Ly6G: neutrophils, Ly6C: monocytes.

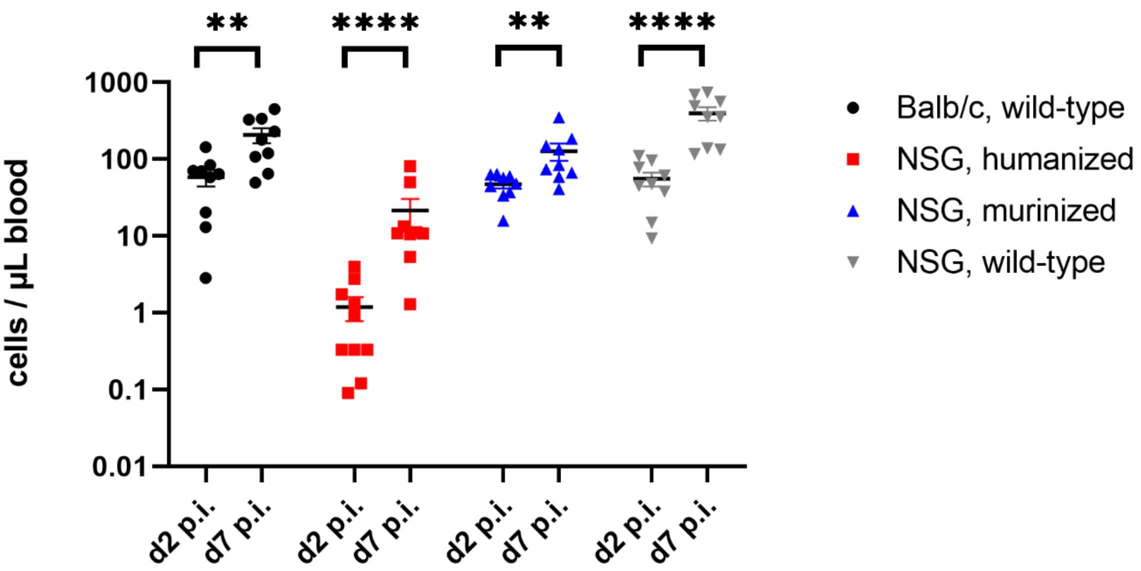

Figure S5: Murine monocytes (mCD45+ Ly6G- Ly6C+) in the blood of infected mice at day 2 or day 7 p.i. analyzed by flow cytometry. Statistical significance was tested with Mann-Whitney-test and p-values displayed (**: $p \leq 0.005$, ****: $p \leq 0.0001$).
